# Untargeted UHPLC-HRMS Metabolomic Profiling of *Cornus mas* L. Fruits: Impact of Conventional and Emerging Extraction Methods on Phenolic Composition and Bioactivity

**DOI:** 10.3390/antiox14121419

**Published:** 2025-11-27

**Authors:** Oleg Frumuzachi, Alexandru Nicolescu, Mihai Babotă, Andrei Mocan, Luigi Lucini, Sascha Rohn, Gianina Crișan, Gabriele Rocchetti

**Affiliations:** 1 Department of Pharmaceutical Botany, “Iuliu Hațieganu” University of Medicine and Pharmacy, 400337 Cluj-Napoca, Romania; oleg.frumuzachi@elearn.umfcluj.ro (O.F.); gcrisan@umfcluj.ro (G.C.); 2 Laboratory of Chromatography, Institute of Advanced Horticulture Research of Transylvania, University of Agricultural Sciences and Veterinary Medicine, 400372 Cluj-Napoca, Romania; alexandru.nicolescu@usamvcluj.ro; 3 Department of Pharmaceutical Botany, Faculty of Pharmacy, “George Emil Palade” University of Medicine, Pharmacy, Sciences and Technology of Târgu Mureș, 540139 Târgu Mureș, Romania; mihai.babota@umfst.ro; 4 Research Center of Medicinal and Aromatic Plants, “George Emil Palade” University of Medicine, Pharmacy, Sciences and Technology of Târgu Mureș, 540139 Târgu Mureș, Romania; 5 Department for Sustainable Food Process, Università Cattolica del Sacro Cuore, Via Emilia Parmense 84, 29122 Piacenza, Italy; luigi.lucini@unicatt.it; 6 Department of Food Chemistry and Analysis, Institute of Food Technology and Food Chemistry, Technische Universität Berlin, Gustav-Meyer-Allee 25, 13355 Berlin, Germany; rohn@tu-berlin.de; 7 Department of Animal Science, Food and Nutrition, Università Cattolica del Sacro Cuore, Via Emilia Parmense 84, 29122 Piacenza, Italy; gabriele.rocchetti@unicatt.it

**Keywords:** metabolomics, spectral annotation, phytochemical characterization, bioactive potential, anthocyanins, iridoids

## Abstract

A growing number of studies evaluated cornelian cherry (*Cornus mas* L.) fruits due to their rich phenolic profile. It is obvious that the efficient recovery of these compounds depends on the extraction method used. Thus, this study aimed at comparing conventional (maceration, decoction) and non-conventional (ultrasound-assisted [UAE], enzyme-assisted [EAE]) extractions. Two novel aspects were characterized: the first application of EAE for cornelian cherry fruit phenolic recovery and the use of untargeted UHPLC-HRMS metabolomics. Untargeted UHPLC-HRMS profiling in positive electrospray ionization mode annotated 342 compounds, the broadest chemical analysis of *C. mas* fruits reported to date, followed by a semi-quantitative assessment of main phenolic compound subclasses using representative standards and multivariate statistical approaches. UAE proved most effective for recovering total flavonoids (433.6 μg eq./g) and phenolic acids (420.2 μg eq./g), while EAE selectively enriched other phenolic category compounds, semi-quantified as oleuropein equivalents (1975.4 μg eq./g). Multivariate analysis further confirmed distinct phytochemical fingerprints across extraction methods: the UAE extract was characterized by isocoumarin 2,4-di-*O*-methylolivetonide, while two compounds, cardamonin and ellagic acid arabinoside, were specifically up-accumulated in the EAE extract. All extracts showed moderate antioxidant (UAE > decoction > maceration > EAE) and enzyme inhibitory activities, with UAE and maceration being the most promising for multifunctional bioactivity. However, while untargeted UHPLC-HRMS revealed unprecedented complexity, results remain tentative and highlight the need for future targeted validation.

## 1. Introduction

**Figure 1:**
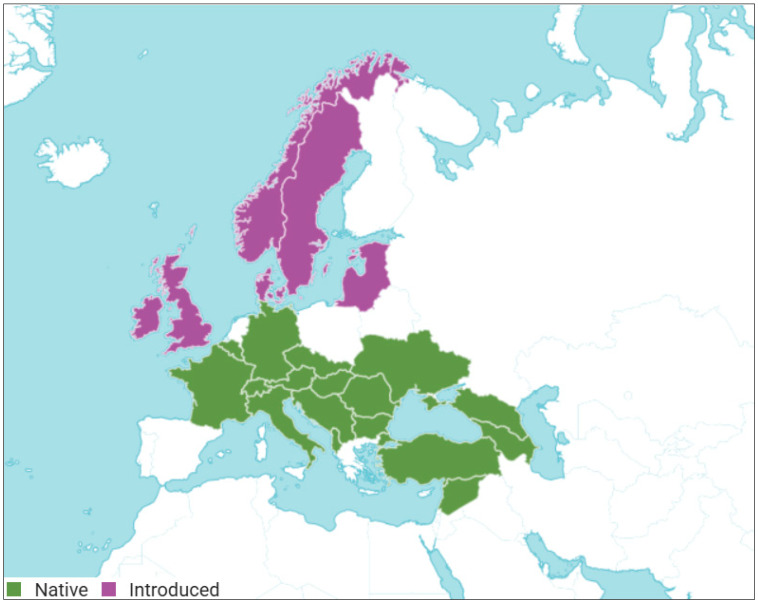
Distribution of *Cornus mas* L. (native and introduced). Adapted from *Cornus mas* L., Plants of the World Online, © Royal Botanic Gardens, Kew, licensed under CC BY 3.0. Source: https://powo.science.kew.org/taxon/urn:lsid:ipni.org:names:271612-1 (accessed on 26 November 2025). Changes were made to the original map.

*Cornus mas* L., commonly known as cornelian cherry, is a species belonging to the genus *Cornus* L. in the Cornaceae family. This deciduous shrub or small tree blooms before leaf development and is characterized by distinctive morphological features, including umbellate cymes terminal subtended by four not showy scale-like bracts, red or purple-black fruits, and a fruit stone wall riddled with cavities [1]. Native to Southern, Central, and Eastern Europe, including countries such as Romania, Moldova, and Ukraine, and Southwest Asia (Turkey, Lebanon–Syria, and Transcaucasus) (Figure 1), *C. mas* has an intense history of traditional medicinal use, dating back to ancient times [2,3]. Ethnopharmacological studies highlight the extensive use of *C. mas* in various regions within its geographical distribution [4]. The fruits of this plant are used in traditional medicine for a wide range of diseases and complaints, with preparations from *C. mas* being considered astringent, tonic, and antipyretic remedies [5].

Recent studies highlighted several key properties of its fruits. One of the prominent characteristics of cornelian cherry fruits is their remarkable antioxidant activity [6]. The antioxidant potential of *C. mas* fruits may be significant in combating oxidative stress, a factor implicated in various degenerative diseases, including severe and ubiquitous cases, such as cancer, diabetes, and cardiovascular issues [7,8,9]. In addition to its antioxidant potential, cornelian cherry exhibits anti-inflammatory properties [10]. Human trials with *C. mas* fruit extract/powder validated some of its health benefits. Aryaeian et al. [11] found that cornelian cherry supplementation has positive effects on bone health and anti-inflammatory processes, Yarhosseini et al. [12] reported statistically lower blood pressure and reduced body fat following cornelian cherry supplementation, while recent meta-analyses concluded that supplementation with cornelian cherry may impact diverse cardiometabolic risk factors (such as anthropometric, glycaemic, and lipid parameters) among individuals considered to be at a high risk [13,14,15].

*C. mas* fruits are distinguished by their rich phytochemical composition, featuring prominent secondary metabolites [16]. The presence of iridoids, including compounds like loganin, loganic acid, sweroside, and cornuside, is associated with different biological activities [17,18,19]. Additionally, cornelian cherry fruits contain different phenolic compounds, including anthocyanins, flavonoids, and phenolic acids [20]. Notable anthocyanins are pelargonidin 3-*O*-galactoside, cyanidin 3-*O*-galactoside, and delphinidin 3-*O*-galactoside [21]. Flavonols such as quercetin and kaempferol glycosides are also detected in *C. mas* fruits [22]. Moreover, triterpenoids, specifically ursolic acid, and carbohydrates are identified as constituents in cornelian cherry fruits [23].

To fully exploit the phyto-medical potential of these fruits, efficient extraction techniques are essential for recovering bioactive compounds [24]. Conventional extraction methods, including maceration and decoction, are widely used, but they are associated with drawbacks like prolonged extraction time, high temperature, energy consumption, and solvent wastage [25]. In response to these limitations, non-conventional extraction methods, such as ultrasound-assisted extraction (UAE) and enzyme-assisted extraction (EAE), emerged as more efficient alternatives [26,27]. These advanced methods not only offer higher extraction efficiency but can also significantly reduce energy consumption, providing a more gentle and sustainable approach for obtaining high-quality extracts.

Different combinations of solvents were used to extract bioactive compounds from cornelian cherry. For instance, hydroalcoholic extraction uses ethanol or methanol mixed with water, which is particularly effective for a broad spectrum of phenolic compounds like flavonoids and iridoids. Some studies showed that hydroalcoholic extracts correlate strongly with antioxidant capacity and have a high efficacy in inhibiting cancer cell proliferation, suggesting a preserved bioactivity [28,29]. On the other hand, simple aqueous extraction with water can yield a substantial total phenolic content (TPC) with strong antioxidant potential [30]. Additionally, beyond the choice of solvent, the extraction method itself also influences the yield of phenolic compounds. For instance, the UAE of anthocyanins from cornelian cherry yielded a high anthocyanin content of 344 mg cyanidin-3-glucoside (Cy3gl) per 100 g of dry weight [31]. In contrast, maceration resulted in a much lower yield, with 14.4 mg Cy3gl per 100 mL [32]. However, these disparities highlight the need for further systematic studies to refine extraction techniques and achieve optimal recovery of bioactive compounds.

As stated earlier, EAE is an innovative and efficient alternative extraction technique, which increases phenolic yield under mild conditions, yet its application remains limited [27]. Previous studies applied EAE to isolate polysaccharides from *Cornus officinalis* Siebold & Zucc. fruits [33,34,35] or to extract oil from *C. wilsoniana* Wangerin fruits [36]; however, the phenolic content of these extracts was not analyzed, and this method has not yet been applied to *C. mas* fruits, highlighting the need to evaluate its potential for phenolic compound recovery.

To overcome the knowledge gaps mentioned (namely, i. the absence of studies applying EAE to *C. mas* fruits; ii. the lack of comparative evaluations of traditional and modern extraction techniques; and iii. the complete unavailability of ultra-high-performance liquid chromatography coupled with high-resolution mass spectrometry [UHPLC-HRMS] metabolomic data describing extraction-dependent differences), the present study aimed to evaluate the potential of aqueous EAE for phenolic compound recovery through a systematic comparison with hydroalcoholic UAE, maceration, and aqueous decoction. This study aims to evaluate the selective fractionation of phenolic compounds using UHPLC-HRMS and the in vitro antioxidant and enzyme inhibitory potential of resultant extracts. By integrating different extraction methods, untargeted UHPLC-HRMS profiling with semi-quantitative class-level analysis, and bioactivity assays, we hypothesized that extraction techniques strongly shape the qualitative and quantitative phenolic profile of *C. mas* fruits, and that modern approaches, particularly UAE and EA, increase the diversity and yield of recovered phenolics compared with traditional methods.

## 2. Materials and Methods

### 2.1. Reagents

The following reagents and standards were utilized in this study: Folin–Ciocalteu reagent, gallic acid, sodium carbonate, rutin, aluminum chloride, ABTS (2,2’-azino-bis(3-ethylbenzothiazoline-6-sulfonic acid)), acetic acid, DPPH (2,2-Diphenyl-1-picrylhydrazyl), ferric chloride hexahydrate, hydrochloric acid, potassium persulfate, sodium acetate, 2,4,6-tris(2-pyridyl)-S-triazine, trolox, 2-amino-2-(hydroxymethyl)-1,3-propanediol (Tris base) (TRIS-RO), 3,5-Dinitrosalicylic acid, 4-nitrophenyl-β-D-glucopyranoside (p-NPG), acarbose, α-amylase from porcine pancreas (11 units/mg solid), α-D-glucoside glucohydrolase from *Saccharomyces cerevisiae* (24 units/mg protein), dimethyl sulfoxide (DMSO), lipase from porcine pancreas (90 units/mg protein), pectinase from *Rhizopus* sp. (412 units/g solid), hemicellulose from *Aspergillus niger* (1.5 units/mg solid), and cellulase (1.8 units/mg solid). All reagents and standards were purchased from Sigma-Aldrich Chemie GmbH (Taufkirchen, Germany).

For UHPLC-HRMS analysis, ethanol and acetonitrile were LC-MS grade, purchased from Sigma-Aldrich S.r.l. (Milan, Italy), while standards for the quantification of phenolic compound classes (ferulic acid, quercetin, catechin, cyanidin, luteolin, resveratrol, oleuropein were purchased from Extrasynthese S.A. (Genay, France).

### 2.2. Plant Material

Ripe fruits of cornelian cherry (*Cornus mas* L.), displaying a deep cherry-red color upon reaching full maturity, were collected in September 2022 from Mereșeni, Hîncești County, Republic of Moldova (46°47′51″ N 28°32′25″ E), and assigned voucher specimen No. 66.1.1.1/09.2022. The harvested plant material underwent sorting, was authenticated based on its botanical characteristics, and was subsequently stored at −20 °C until the subsequent extraction phase.

### 2.3. Extraction of Bioactive Compounds

Initially, the removal of stones from cornelian cherry fruits was performed manually using a knife, followed by the manual grinding of the flesh and skin using a mortar and pestle until a paste consistency was achieved. Each experimental configuration involved a specific extraction method: Decoction was carried out by boiling 20 g of cornelian cherry fruits in 180 mL of distilled water for 15 min at 100 °C. According to the pharmacopeial requirements (*Ph.Eur. 10.8, Romanian Pharmacopoeia 10th ed*), for maceration, 20 g of fruits were soaked in 180 mL of 70% ethanol, keeping the mixture at room temperature in the dark for 10 days. The mixture was kept in a sealed 250 mL Duran bottle to minimize ethanol evaporation and was manually mixed once daily [37]. UAE utilized 20 g of fruits in 180 mL of 70% ethanol, subjected to 60 min of extraction using a standard ultrasound bath (230 V, 50 Hz, 310 W, Polsonic Palczynski Sp. J, Warsaw, Poland) [38]. EAE employed a slightly modified method previously described by Nicolescu et al. [39]. It involved adding 20 g of cornelian cherry fruits to 180 mL of distilled water, which contained an enzymatic blend consisting of pectinase, hemicellulase, and cellulase in a ratio of 1:2:2 based on enzymatic activity, respectively. The final enzymatic activities in the extraction mixture were 0.6 U/mL (pectinase), 0.3 U/mL (hemicellulase), and 0.3 U/mL (cellulase), referring to the final activity per mL of extraction mixture and calculated according to the activity specifications provided by the manufacturer. The mixture was incubated at 50 °C for 60 min, with constant stirring at 500 rpm using an Arec.x Heating Magnetic Stirrer (VELP Scientifica S.r.l., Usmate Velate MB, Italy).

Each extraction method was performed using three independent biological replicates, each derived from a distinct fruit batch. After extraction, the three biological replicates corresponding to each method were combined into a single pooled extract, which was then vacuum filtered to eliminate any solid residues. Afterwards, the ethanol was removed by evaporation utilizing a rotary evaporator (AC 230 V, 50 Hz, 1145 W, Heidolph 94200, Heidolph Scientific Products GmbH, Schwabach, Germany), under reduced pressure at 30 °C, and the resulting extracts were subjected to freeze-drying using the Vertical Freeze Dryer BK-FD18S (Biobase International Co. Ltd., Jinan, China), being then stored in a desiccator at room temperature for subsequent analysis. The obtained extracts were designated as follows: **CME**—EAE extract, **CMF**—decoction extract, **CMM**—maceration extract, **CMU**—UAE extract. Consequently, all subsequent analyses (TPC, TFC, UHPLC-HRMS, antioxidant assays, and enzyme-inhibition assays) were conducted using technical triplicates of the same lyophilized pooled extract.

The traditional extraction approaches sought to replicate the conventional techniques used in Romania for crafting traditional beverages like compote (through decoction) and cornelian cherry fruit liqueur (via maceration). In contrast, the modern methods were used to investigate advanced and more effective extraction techniques, including UAE and EAE. The goal was to improve the overall extraction efficiency and quality of bioactive compounds derived from *C. mas* fruits by employing these innovative and modern approaches.

### 2.4. Total Phenolic Content (TPC) and Total Flavonoid Content (TFC)

The initial evaluation of TPC involved the adaptation of the Folin–Ciocalteu method for a SPECTROstar Nano microplate reader (BMG Labtech GmbH, Ortenberg, Germany) [40]. In brief, triplicate diluted samples (20 µL) were mixed with diluted Folin–Ciocalteu reagent (100 µL, 1:9, *v*/*v*) and vigorously shaken. After 3 min, a 1% Na_2_CO_3_ solution (80 µL) was added and following a 30 min incubation at room temperature, absorbance was measured at 760 nm. The results were expressed as milligrams of gallic acid equivalents per g of freeze-dried extract (mg GAE/g extract). TFC determination utilized the aluminum chloride method [40]. In a 96-well plate, 100 μL of the sample was mixed with 100 μL of a 2% AlCl_3_ aqueous solution. After a 15 min incubation in the dark at room temperature, absorbance was measured at 420 nm using the microplate reader. Results were presented in milligrams of quercetin equivalents per gram of freeze-dried extract (mg QE/g extract).

### 2.5. UHPLC-HRMS Analysis of Phenolic Profile

The lyophilized *C. mas* fruit extracts (150 mg) were dissolved in 1.5 mL of the proper extraction solvent (namely water for CMF and CME, 70% ethanol for CMM and CMU) and then centrifuged at 6000× *g* for 10 min at 4 °C. The obtained supernatants were filtered using 0.22 cellulose syringe filters and analyzed for untargeted phenolic profiling. This latter was performed by HRMS using a Q-Exactive™ Focus Hybrid Quadrupole-Orbitrap Mass Spectrometer (Thermo Scientific Inc., Waltham, MA, USA) coupled to a Vanquish ultra-high-pressure liquid chromatograph (UHPLC) of the same supplier. All the details regarding UHPLC-MS/MS analysis are described in Babotă et al. [41]. Chromatographic separation was performed using a water–acetonitrile gradient (0.1% formic acid in both LC-MS grade phases), increasing acetonitrile from 6% to 94% over 35 min on an Agilent Zorbax Eclipse Plus C18 column (50 × 2.1 mm, 1.8 μm). The flow rate was 200 μL/min, with a 6 μL injection volume. Data were acquired in full-scan mode (*m*/*z* 100–1200, positive ionization, 70,000 FWHM; AGC 1 × 10^6^; IT 200 ms) coupled with data-dependent MS/MS (Top *N = 3*). In DDA mode, resolution was set to 17,500 at *m*/*z* 200, with AGC 1 × 10^5^, IT 100 ms, and a 1.0 *m*/*z* isolation window. Precursor ions were fragmented using a normalized collision energy of 23.3 eV. Data quality was ensured through blank subtraction (solvent only) to remove background signals and through LOWESS-based drift correction implemented in the software MS-DIAL (version 4.90). Under these experimental conditions, a level 2 of confidence in annotation was reached via spectral matching against the comprehensive MSMS ESI(+) database provided by MS-DIAL and the Phenol-Explorer database [42], using a tolerance for mass accuracy of 5 ppm. Metabolite annotation was performed considering the most common adducts observed under our ESI+ conditions, namely [M + H]^+^, [M + Na]^+^, [M + ACN + H]^+^, and [M − H_2_O + H]^+^, based on the acidified water/acetonitrile solvent system. Peak-filling algorithm with a 5 ppm tolerance was applied to address missing data. Finally, all the phenolic compounds identified were grouped into different typical subclasses and then semi-quantified according to hydroalcoholic standard solutions of pure standard compounds analyzed under the same instrumental conditions. Overall, seven phenolic compounds, namely ferulic acid (phenolic acids), quercetin (flavonols), catechin (flavan-3-ols), cyanidin (anthocyanins), and luteolin (flavones and other remaining flavonoids), resveratrol (stilbenes), oleuropein (other remaining phenolics), were used for this purpose. The semi-quantitative results were expressed as μg of equivalents (Eq.)/g lyophilized extract (DM) (*n* = 3).

### 2.6. In Vitro Assays of Antioxidant Capacity

To evaluate the in vitro antioxidant capacity of the four extracts, three complementary assays were employed, as outlined in recent studies [40,43]. The ABTS assay served as an indicator of radical scavenging activity. For this, an ABTS^+^ radical solution was prepared by combining a 7 mM ABTS solution with 2.45 mM potassium persulfate, left in the dark at room temperature for 16 h. The resulting solution was diluted to achieve an absorbance of 0.70 ± 0.02 at 734 nm. Subsequently, 200 µL of the radical solution was added to 20 µL of the sample (at a concentration of 1.5 mg/mL). After a 30 min incubation in the dark at room temperature, absorbances were measured at 734 nm, and the results were expressed as milligrams of Trolox equivalents per gram of freeze-dried extract (mg TE/g dw).

In the DPPH assay, 30 μL of the sample (at a concentration of 1.25 mg/mL) was mixed with 270 μL of a 0.004% methanol solution of DPPH. Following a 30 min incubation at room temperature in the dark, absorbance was measured at 517 nm, and the DPPH radical scavenging activity was quantified, expressed as milligrams of Trolox equivalents per gram of freeze-dried extract (mg TE/g extract).

For the FRAP assay, the FRAP reagent was prepared by combining acetate buffer (0.3 M, pH 3.6), 2,4,6-tris(2-pyridyl)-S-triazine (TPTZ) (10 mM), 40 mM HCl, and ferric chloride (20 mM) in a ratio of 10:1:1 (*v*/*v*/*v*). Subsequently, 175 µL of the radical solution was added to 25 µL of the sample (at a concentration of 1 mg/mL), and absorbance was measured at 593 nm after a 30 min incubation at room temperature in the dark. Antioxidant activity was expressed as milligrams of Trolox equivalents per gram of freeze-dried extract (mg TE/g extract).

### 2.7. Enzyme Inhibitory Activity

The inhibitory effects of four extracts were evaluated against acetylcholinesterase, *α*-glucosidase, *α*-amylase, tyrosinase, and pancreatic lipase utilizing various in vitro assays [44,45,46,47]. For the α-glucosidase inhibition assay, 50 µL of extracts at different dilutions were mixed with 50 µL of the enzyme (in phosphate buffer at pH 6.8). The reaction mixture was incubated at 37 °C for 15 min. Subsequently, 50 µL of the substrate (10 mM p-nitrophenyl- α-D-glucoside in phosphate buffer) was added, and the reaction mixture was incubated again at 37 °C for 10 min. Finally, absorbance was measured at 400 nm. Acarbose was used as a positive control, and IC_50_ values (µg/mL) were calculated.

The assessment of inhibitory activity against alpha-amylase was conducted using the Caraway Somogyi iodine/potassium iodide (IKI) method. To prepare the IKI solution, 5 mM of I_2_ was dissolved in 5 mL of DMSO, and 5 mM of KI was dissolved in 5 mL of sodium phosphate buffer (20 mM, pH 6.9 with 6 mM sodium chloride). Subsequently, these solutions were combined, and the final volume was adjusted to 100 mL by adding sodium phosphate buffer. In a 96-well microplate, a sample solution (25 µL) was mixed with amylase solution (50 µL, 0.05 mg/mL) in phosphate buffer and pre-incubated for 10 min at 37 °C. After pre-incubation, the reaction was initiated by adding starch solution (50 µL, 0.05%). The reaction mixture was incubated for 10 min at 37 °C. Subsequently, the reaction was stopped with the addition of HCl (25 µL, 0.1 M), followed by the addition of IKI solution (100 µL). Absorbance readings were taken at 615 nm, and IC_50_ values (µg/mL) were calculated using acarbose as a positive control.

For the pancreatic lipase inhibition assay, a solvent for the enzyme was prepared by combining 100 mM Tris-HCl and 5 mM CaCl_2_ at pH 7.0. Subsequently, 40 µL of this solution was mixed with 40 µL of extracts at various dilutions and incubated in the dark at 37 °C for 15 min. Afterward, 20 µL of p-nitrophenyl butyrate (p-NPB), the substrate, was added, and the mixture was further incubated for 15 min under the same conditions. Lipase inhibition activity was determined by measuring absorbance at 405 nm, and IC_50_ values (µg/mL) were calculated using orlistat as a positive control.

For assessing the tyrosinase inhibition activity, 40 μL of sample solutions at various concentrations were combined with 80 μL of potassium phosphate buffer (50 mM, pH = 6.5) and 40 μL of tyrosinase enzyme solution (125 U/mL) in a 96-well plate. The resulting mixture underwent pre-incubation for 5 min at 37 °C. Following this, 40 µL of L-3,4-dihydroxyphenylalanine (10 mM) was added, and the newly formed mixture was incubated for an additional 15 min. Finally, the absorbance of the reaction mixture was measured at 492 nm, with results presented as IC_50_ values (μg/mL). Kojic acid served as a positive control.

Lastly, a microplate reader-adapted protocol was employed to assess the acetylcholinesterase inhibition capacity of the extracts. In this adapted procedure, 25 μL of sample solutions at different concentrations were combined with 50 μL of Tris-HCl buffer (50 mM, pH = 8), 125 μL of DTNB (0.9 mM), and 25 μL of acetylcholinesterase enzyme solution (0.078 U/mL). The reaction mixture underwent pre-incubation for 15 min at 37 °C. Following this, 25 μL acetylthiocholine iodide (4.5 mM) was added to the reaction mixture, and the plate was incubated for an additional 10 min at 37 °C. Ultimately, the absorbance was measured at 405 nm, and the results were expressed as IC_50_ values (μg/mL). Galantamine served as a positive control.

### 2.8. Statistical and Correlation Analysis

Each extract was tested in technical triplicate (as per Section 2.3). Statistical analysis utilized the GraphPad Prism 9 program, with significance differences determined at the α = 0.05 level using one-way analysis of variance (ANOVA) followed by Tukey’s HSD. All data were presented as mean values with standard deviations (mean ± SD).

## 3. Results

### 3.1. Cornelian Cherry Fruit Extracts Phytochemical Analysis

**Figure 2:**
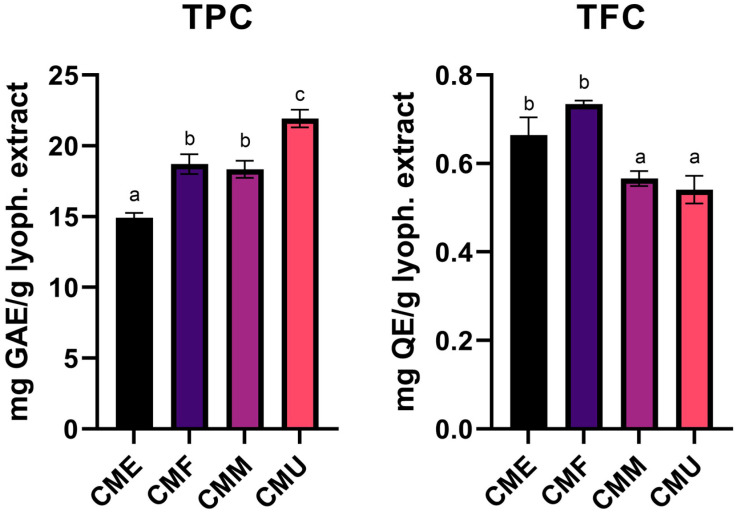
Comparison of total phenolic (TPC, milligrams of gallic acid equivalents per g of lyophilized extract [mg GAE/g extract]) and total flavonoid (TFC, milligrams of quercetin equivalents per gram of lyophilized extract [mg QE/g extract]) contents in *Cornus mas* fruit extracts obtained by enzyme-assisted (CME), decoction (CMF), maceration (CMM), and ultrasound-assisted (CMU) extraction. Values represent mean ± standard deviation (SD) of triplicates (*n* = 3). Different letters indicate statistically significant differences (*p* < 0.05).

Before the evaluation of the bioactive properties of the cornelian cherry extracts obtained through different extraction methods, an initial chemical characterization was performed. Preliminary results (Figure 2) showed TPC values ranging from 14.9 to 21.9 mg GAE/g extract in CME and CMU extracts, while TFC values ranged from 0.66 to 0.73 mg QE/g extract in CME and CMF extracts.

The untargeted UHPLC-HRMS profiling carried out in this study revealed a remarkable diversity of compounds in *C. mas* fruit extracts, with a total of 342 annotations reported in the Supplementary Materials. It included the most cited compound identified in cornelian cherry fruits, such as cyanidin 3-*O*-glucoside, malvidin 3-*O*-galactoside, pelargonidin 3-*O*-arabinoside, quercetin, ellagic acid, caffeic acid, and rosmarinic acid. The identified compounds mostly belong to the flavonoid class, specifically phenolic acids, flavonols, anthocyanins, and flavan-3-ols.

To provide a clearer picture of the distribution of phenolic subclasses within the extracts, a semi-quantitative class analysis based on representative standards was carried out, the results of which are presented in Table 1.

Significant differences were measured when considering the different phenolic compound classes in CME, CMF, CMM, and CMU. Overall, CMU was found to be the richest source of total flavonoids, including anthocyanins, flavanols, flavonols, and flavones, reaching a cumulative content of 433.6 μg eq./g, thereby confirming the strong ability of ultrasound treatment to enhance the extraction of this phenolic class from *C. mas* fruits. A similar trend was found when considering the semi-quantitative distribution of phenolic acids; in this regard, as shown in Table 1, CMU was identified as the best source (420.2 μg eq./g), followed by CMM (263.1 μg eq./g). As far as the phenolic class of stilbens is concerned, no significant differences were measured when comparing the different extraction techniques, recording an average value of ~100 μg eq./g. Finally, a higher effectiveness of the EAE (CME) in promoting the recovery of other phenolics (quantified as oleuropein equivalents) was measured, recording a significantly higher semi-quantitative content (1975.4 μg oleuropein eq./g; Table 1) when compared with the other extraction systems, being particularly rich in caftaric acid and pyrocatechol (Supplementary Materials).

### 3.2. Multivariate Discrimination of Extraction Methods

**Figure 3:**
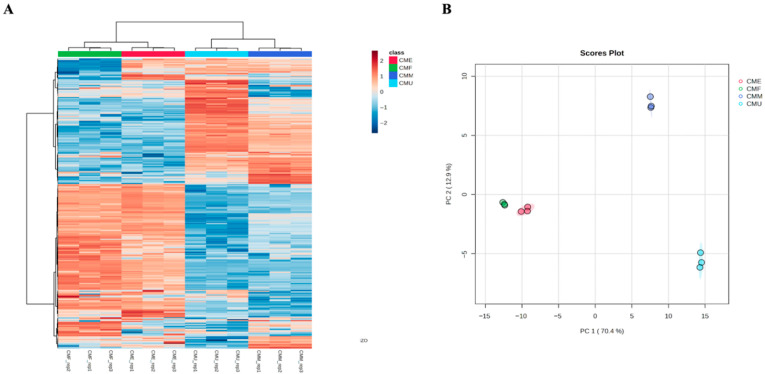
Unsupervised statistical findings from both hierarchical clustering heat map (**A**) and principal component analysis score plot (**B**). **CME**—enzyme-assisted extraction extract; **CMF**—decoction extract; **CMM**—maceration extract; **CMU**—ultrasound-assisted extraction extract.

The Supplementary dataset is not presented merely as a compound list but as evidence that extraction conditions diversify metabolite recovery. These differences were further examined through the application of unsupervised multivariate approaches. Hierarchical clustering (Figure 3A) and principal component analysis (Figure 3B) consistently separated the extracts into two groups: CME and CMF on one side, and CMM and CMU on the other. The first two principal components explained 83.3% of the variance, confirming that phenolic profiles alone are sufficient to discriminate between extraction methods. This grouping highlights that UAE and maceration tend to share similar phytochemical outputs enriched in flavonoid glycosides, whereas EAE and decoction cluster together through the accumulation of hydrolyzed phenolics.

### 3.3. Identification of Discriminant Markers by OPLS-DA

Building on these exploratory results, supervised OPLS-DA combined with VIP selection was subsequently applied to identify the compounds most responsible for the observed separations (Supplementary Materials). The model showed excellent reliability, with R^2^ = 0.997 and Q^2^ = 0.979. In total, 147 phenolic compounds had VIP scores > 1, indicating predictive relevance. The most discriminant markers (VIP > 1.6) revealed clear contrasts between techniques: ten compounds were strongly enriched in CMU extract, including the isocoumarin 2,4-di-*O*-methylolivetonide (the highest VIP marker), while two compounds, cardamonin and ellagic acid arabinoside, were specifically up-accumulated in CME extract. These findings confirm that ultrasound promoted the recovery of phenolic compounds that were uniquely detected or markedly more abundant in the UAE extract (e.g., 2,4-di-*O*-methylolivetonide), while enzymatic treatment favored a narrower set of hydrolyzed derivatives. Beyond reinforcing the PCA clustering, these discriminant markers may serve as authenticity fingerprints to differentiate extraction methods and ensure reproducibility in future applications.

### 3.4. Antioxidant Activity of Cornelian Cherry Fruit Extracts

**Figure 4:**
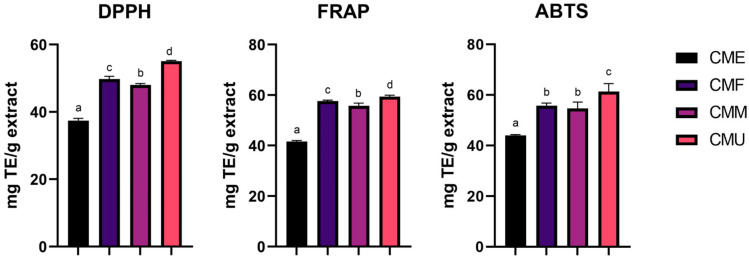
In the in vitro assessment of antioxidant activity in cornelian cherry fruit extracts, expressed as milligrams of Trolox equivalents per gram of freeze-dried extract (mg TE/g extract). Values are expressed as mean ± standard deviation (SD) of triplicates (*n* = 3). Different letters above the bars denote statistically significant differences among extracts (*p* < 0.05). **CME**—enzyme-assisted extraction extract; **CMF**—decoction extract; **CMM**—maceration extract; **CMU**—ultrasound-assisted extraction extract.

In this study, ABTS, DPPH, and FRAP assays, known for their broad application in phenolic compound analysis, were employed for assessing the antioxidant capacity of the evaluated extracts. These assays are valued for their convenience, high-throughput capabilities, and independence from specialized equipment, operating as “fixed-time” assays with reaction durations ranging from 10 to 30 min. As depicted in Figure 4, the CMU extract exhibited the highest antioxidant activity, with the CME extract displaying the lowest antioxidant capacity. While the results demonstrate statistical significance in all assays (except for the ABTS assay, where there is no statistically significant difference between CMF and CMM extracts), the absolute variances between CMF, CMM, and CMU extracts are relatively modest.

### 3.5. Assessment of the In Vitro Anti-Enzymatic Activity of Extracts from Cornelian Cherry Fruit Extracts

**Figure 5:**
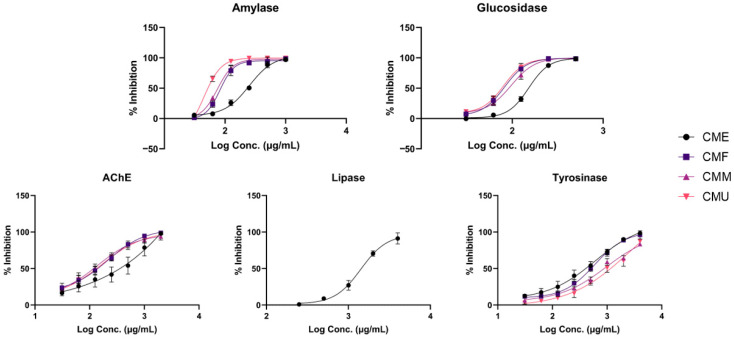
The concentration-dependent enzyme-inhibitory activity (expressed as logC, in μg/mL) of *Cornus mas* fruit extracts. Values represent mean ± standard deviation (SD) of triplicates (*n* = 3). CME—enzyme-assisted extraction extract; CMF—decoction extract; CMM—maceration extract; CMU—ultrasound-assisted extraction extract.

The inhibitory effects of four extracts from cornelian cherry fruits were evaluated against *α*-glucosidase, *α*-amylase, pancreatic lipase, acetylcholinesterase, and tyrosinase using diverse in vitro methods, aiming to elucidate their potential antidiabetic, anti-obesity, neuroprotective, and dermatological benefits. In this investigation, acarbose, a commercial inhibitor of *α*-glucosidase and *α*-amylase, exhibited IC_50_ values of 48.15 and 19.37 μg/mL, respectively. In comparison, cornelian cherry extracts demonstrated lower efficacy in inhibiting α-glucosidase activity, with IC_50_ values ranging from 77.2 (CMU) to 151.4 μg/mL (CME), as outlined in Figure 5 and Table 2. Similarly, relative to acarbose, cornelian cherry extracts were less potent in inhibiting α-amylase activity, with IC_50_ values spanning from 52.5 (CMU) to 233.9 μg/mL (CME). On the other hand, a moderate inhibition of lipase was observed only in the case of the CME extract, exhibiting an IC_50_ value of 1434.7 μg/mL. The other extracts demonstrated an inhibition percentage of approximately 55% at a concentration of 4000 μg/mL. Additionally, the inhibition of acetylcholinesterase was also moderate, with IC_50_ values spanning from 118.4 (CMM) to 280.8 μg/mL (CME). Similarly, the inhibition of tyrosinase was relatively low, with IC_50_ values ranging from 406.3 (CME) to 1184.2 μg/mL (CMU).

### 3.6. Correlation Coefficients Between Bioactivities and Phenolics

Finally, the phenolic class measured by the semi-quantitative HRMS approach was correlated to the different bioactivities by evaluating Pearson’s correlation coefficients. The results are summarized in Table 3, considering only the phenolic classes establishing at least one significant correlation with one bioactivity measured (*p* < 0.05). As a general consideration, our findings showed that the most explained bioactivity was the tyrosinase inhibition of the different *C. mas* extracts, with flavonols, phenolic acids, flavanols, and anthocyanins establishing very strong and positive correlations. Another interesting piece of information was related to the antioxidant activity of the extracts; in this regard, DPPH was the most explained bioactivity, mainly associated with flavonoids (both flavanols and flavonols) and phenolic acids. Finally, the class of other phenolic compounds (semi-quantified as oleuropein equivalents) was the only one establishing significant correlations with the amylase and glucosidase inhibition (Table 3).

## 4. Discussion

### 4.1. Phytochemical Variability Observed in Cornelian Cherry Fruit Extracts

Phenolic compounds are key compounds in the analytical characterization of plant secondary metabolites. In this study, TPC and TFC assays provided rapid estimates of phenolic compounds, being complemented by exhaustive UHPLC-HRMS profiling. Despite their non-specificity, these colorimetric methods remain valuable for preliminary screening and contextualization of metabolomic data [48]. Previous studies consistently describe *C. mas* fruits as rich in phenolic compounds, although reported values vary widely across genotypes and extraction methods. For example, Ukrainian cultivars showed TPC ranges from 100.7 to 924.7 mg GAE·100 g^−1^ FW [29], while Romanian genotypes displayed substantially higher levels, with TPC between 910 and 2330 mg GAE·100 g^−1^ extract and TFC ranging from 50 to 160 mg QE·100 g^−1^ extract [49].

Compared with earlier targeted analytical approaches (HPLC-DAD, HPLC-MS), which typically identified only 10–20 compounds per study [18,19,50], the untargeted UHPLC-HRMS analysis applied herein provided a considerably broader chemical overview. Unlike targeted methods that predefine analytes and depend on available standards, untargeted profiling captures the widest possible range of metabolites based on accurate mass, retention time, and MS/MS fragmentation patterns [51]. Earlier works mainly reported iridoids (e.g., loganic acid, loganin), a limited set of anthocyanins (e.g., cyanidin-3-galactoside), and several quercetin- and kaempferol-based flavonol glycosides. Even a comprehensive literature review summarized only ~50 compounds identified across different studies [52]. In contrast, our UHPLC-HRMS approach enabled the annotation of a much larger and chemically diverse set of phenolic compounds, highlighting the advantage of untargeted metabolomics in revealing known and previously unreported compounds.

The high resolution of Orbitrap mass spectrometry, combined with advanced data processing (MS-DIAL spectral matching and Phenol-Explorer database integration), enabled a much wider chemical profile. Orbitrap HRMS offers significantly higher resolution and sensitivity than triple-quadrupole or ion-trap MS used in earlier studies [53]. Thus, the present dataset complements previous literature, suggesting that cornelian cherry fruits might contain a more complex phytochemical profile than previously reported. For instance, the dataset presented herein included tentative annotations of several compounds not previously reported in *C. mas*. These include 8-*O*-acetyl shanzhiside methyl ester, cardamonin, ellagic acid arabinoside, and isocoumarin derivatives such as 2,4-di-*O*-methylolivetonide. The tentative annotation of cardamonin, a chalconoid usually found in Alpinia and Boesenbergia species [54], is intriguing but requires targeted confirmation. If validated, these findings could point to previously overlooked metabolic pathways or compound classes in cornelian cherry, or alternatively, reflect spectral similarity misannotations. However, the presented results should serve as hypotheses for further targeted studies rather than definitive identifications.

Perhaps the most striking discrepancy between the presented findings and earlier studies is the apparent absence of loganic acid, which is frequently reported as the dominant iridoid in *C. mas* fruits. Numerous chromatographic separations consistently list loganic acid as a major marker compound [21,52,55]. The absence of it might be explained by the method employed. Loganic acid is highly polar and tends to ionize inefficiently in positive ESI mode, which was applied in our untargeted analysis, whereas previous studies often relied on negative ionization or derivatization strategies more favorable for small polar glycosides [17,18,56]. Under these conditions, loganin was more readily detected, as its esterified form is less polar and chemically more stable. The simultaneous absence of loganic acid and presence of loganin in the presented datasets may therefore reflect the combined influence of ionization mode, compound stability during extraction, and matrix-related factors. In this regard, ion suppression is a main challenge in complex fruit extracts, where abundant anthocyanins and phenolic acids co-elute, and additional handling steps such as freeze-drying and re-dissolution may have promoted partial hydrolysis. Although loganic acid is generally stable, its non-detection here cannot be excluded as a preparation artifact, further underscoring that extraction and analytical conditions critically shape the phytochemical profiles observed.

The semi-quantitative approach, based on representative standards for each class, is not directly comparable with absolute quantifications reported in the literature. Nonetheless, general patterns align: previous studies consistently highlight anthocyanins (pelargonidin- and cyanidin-glycosides), iridoids (loganic acid, loganin, sweroside), and flavonols (quercetin, kaempferol glycosides) as major constituents [21,52,55]. All of these classes were observed, though with different relative intensities. For instance, Aurori et al. [50] reported cyanidin-3-galactoside as dominant among anthocyanins, whereas in the present UAE extracts, multiple cyanidin derivatives were identified (cyanidin 3-*O*-(3″,6″-*O*-dimalonyl-glucoside), cyanidin 3-*O*-(6″-dioxalyl-glucoside), cyanidin 3-*O*-(6″-malonyl-3″-glucosyl-glucoside), cyanidin 3-*O*-galactoside, and cyanidin 3-*O*-xylosyl-rutinoside), likely reflecting higher extraction efficiency and analytical sensitivity. Thus, while individual compound rankings differ, the general phytochemical “signature” of cornelian cherry fruits was preserved.

It should be noted that, in line with the Metabolomics Standards Initiative [57], the compounds reported herein correspond to annotation level 2: putative identifications based on accurate mass and MS/MS spectral similarity with databases. Without validation by authentic standards, these remain tentative identifications rather than confirmed level 1 identifications. The purpose of the current work was to map the metabolomic diversity shaped by different extraction techniques, and the resulting dataset provided a strong basis for future targeted studies. Indeed, similar metabolomics-based analyses in other fruit matrices showed that untargeted profiling often captures far more compounds than classical targeted analysis, although validation is always required for definitive proof [58,59].

### 4.2. Antioxidant Potential of Cornelian Cherry Fruit Extracts and Influence of Extraction Technique

The antioxidant results obtained in this study, where CMU consistently showed the highest DPPH, FRAP, and ABTS values followed by CMF and CMM, with CME exhibiting the lowest activity, highlight clear differences in the ability of the extracts to neutralize free radicals. These findings are relevant given the well-established role of oxidative stress in the pathogenesis of cardiovascular, neurodegenerative, and metabolic diseases [60,61,62].

Previous studies investigated the antioxidant activity of *C. mas* fruit extracts. In the study described by Tenuta et al. [16], extracts from the hydroalcoholic maceration of fresh and dried *C. mas* fruits showed the most significant radical scavenging activity with IC_50_ values of 3.94 and 1.18 μg/mL in the DPPH and ABTS tests, respectively. In contrast, the extract resulted from the UAE of dried *C. mas* fruits showed the most significant antioxidant activity in the FRAP assay, with an IC_50_ value of 9.5 μM Fe(II) per g [16]. Furthermore, Gillani et al. [38] employed various extraction methods to isolate bioactive compounds from cornelian cherry fruits, including UAE with three solvents (water, water/ethanol 50:50 *v*/*v*, and ethanol), supercritical fluid extraction, and subcritical water extraction. In the beta-carotene/linoleic acid test, the water/ethanol (50:50, *v*/*v*) ultrasound-assisted extract exhibited robust antioxidant activity, reaching 85.84%. In the oil oxidative stability index assay, the cornelian cherry extracts displayed superior resistance to oxidation compared to the synthetic antioxidant tert-butylhydroquinone (TBHQ). Notably, the water/ethanol (50:50, *v*/*v*) ultrasound-assisted extract exhibited the lengthiest induction time, lasting 4.50 h, highlighting its remarkable potential in enhancing oxidative stability [38]. Nevertheless, Aurori et al. [50] reported that the antioxidant capacity of an ethanolic extract from cornelian cherry fruits, assessed through DPPH, was 0.5 mg/mL, and the FRAP value was 23 μmol Trolox/mL.

The subtle differences in antioxidant activity between CMF, CMM, and CMU extracts, despite statistical significance, suggest that these methods might be extracting a similar profile of antioxidants. Maceration, decoction, and UAE extracted potential antioxidants far more efficiently than EAE, and several factors contributed to this difference observed. Firstly, the prolonged heat exposure during decoction likely facilitated the extraction of a broad profile of antioxidants, particularly those specifically sensitive to heat. Maceration, involving an extended soaking period in ethanol, effectively extracted water-soluble as well as lipophilic antioxidants present in cornelian cherry fruits. UAE, with its disruptive mechanical forces, contributed to a higher release of antioxidants by disintegrating cell structures. Conversely, EAE (CME extract) may have faced challenges in achieving optimal conditions for antioxidant extraction. Enzymatic activity is influenced by factors like pH, temperature, and the specific enzymes used. In this case, the conditions might not have been advantageous for maximizing the release of antioxidants from the cornelian cherry fruits. However, to the best of current knowledge, this study represents the first characterization exploring the impact of EAE on phenolic compounds from cornelian cherry fruits. Moreover, it is important to note that the choice of extraction method significantly impacts the composition of the obtained extracts, influencing the types and quantities of bioactive compounds extracted. This underlines the importance of selecting an optimum extraction method based on the targeted compounds and desired properties of the final extract.

### 4.3. Comparative Interpretation of Extraction-Dependent Metabolite Patterns

Nonetheless, it is important to contextualize the observed patterns with what is already known about *C. mas* phytochemistry and extraction mechanisms. Cornelian cherry fruits are a well-documented source of phenolic compounds, particularly anthocyanins, flavonols, and phenolic acids, which contribute substantially to their antioxidant activity [2,50]. The recovery of these compounds, however, is strongly influenced by the extraction method, as different conditions affect cell wall disruption and compound stability. For instance, Behrangi et al. [63] compared shaking, ultrasonic, and Soxhlet extractions in *C. mas*, showing that shaking and ultrasound were more efficient than Soxhlet for phenolic compound recovery, with *C. mas* showing high total phenolic content and strong antioxidant activity. Similarly, Dumitrașcu et al. [31] reported that UAE was particularly effective for maximizing anthocyanin and total phenolic recovery from *C. mas*, with optimal yields obtained at 30 °C and 40 min in aqueous ethanol, identifying cyanidin-3-rutinoside and cyanidin-3-glucoside as the main anthocyanins. Their work also demonstrated that anthocyanins are relatively thermo-resistant up to 120 °C, but degrade rapidly at higher temperatures, following first-order kinetics. Finally, Enache et al. [64] compared conventional water extraction and hydroalcoholic UAE and found that these techniques could provide extracts with high recovery of phenolic compounds and antioxidant activity, although optimal conditions differed based on the method. These findings are consistent with the results of the study herein, where UAE enhanced the recovery of phenolic compounds.

Indeed, the UAE is frequently highlighted for its efficiency in releasing phenolic compounds through cavitation, which disrupts plant tissues and promotes solubilization. This method enhances anthocyanin recovery, compounds otherwise prone to degradation at high temperature, while preserving bioactivity due to shorter extraction times [65]. In our study, UAE extract (CMU) was enriched in several distinctive compounds, including epigallocatechin, galangin, isoquercetin, and quercetin-3-*O*-glucosyl-6″-acetate (Supplementary Materials), underlining ultrasound’s ability to promote diverse flavonoid release. Similarly, EAE relies on pectinase, cellulase, and hemicellulase to degrade cell wall polysaccharides, liberating phenolic acids and flavonoid glycosides from otherwise inaccessible complexes [66]. By contrast, maceration, though less efficient and time-consuming, remains capable of extracting substantial amounts of phenolic compounds when organic solvents are used [67], and in our conditions was particularly effective for anthocyanins and low-molecular-weight compounds (Table 1). Decoction, on the other hand, subjects plant material to prolonged high temperatures, which can facilitate the release of certain phenolic acids but at the cost of degrading heat-sensitive flavonoids [68]. Consistent with this, our decoction extract (CMF) yielded comparatively lower phenolic levels, though typical iridoid derivatives such as loganin were better preserved, reflecting their higher thermal stability. Taken together, these observations indicate that while UAE and EAE represent modern approaches that maximize the diversity of recovered compounds, maceration and decoction still play a role in selectively enriching certain stable metabolites.

### 4.4. Enzyme Inhibitory Activities of Cornelian Cherry Fruit Extracts and Their Potential Biological Implications

The assessment encompassed the extracts’ capacity to inhibit α-glucosidase, responsible for breaking down complex carbohydrates; *α*-amylase, facilitating starch digestion; pancreatic lipase, participating in the breakdown of dietary fats; acetylcholinesterase, involved in neurotransmitter regulation; and tyrosinase, catalyzing the conversion of phenolic compounds to brown-pigmented melanin. These enzymes play key roles in carbohydrate and lipid metabolism, making them strategic targets for managing blood glucose and body weight [69]. Moreover, acetylcholinesterase regulates neurotransmitter levels, influencing cognitive function, while tyrosinase is pivotal in melanin synthesis. Modulating these enzymes holds implications for neurodegenerative and skin-related disorders [70,71].

In the characterization of *C. mas* fruit extracts for enzyme inhibition potential, Świerczewska et al. [72] found IC_50_ values of 134 ± 6.4 mg/mL and 92.5 ± 16.6 mg/mL for *α*-amylase inhibition in aqueous and ethanolic extracts, respectively. Conversely, Blagojević et al. [73] reported IC_50_ values ranging from 3560 μg/mL to 1381 μg/mL for *α*-amylase inhibition across different cornelian cherry genotypes, obtained through three successive ultrasound extractions. The activity against *α*-amylase was also assessed by Tenuta et al. [16], resulting in IC_50_ values ranging from 208.2 to 953.6 μg/mL. The inhibitory potential against *α*-glucosidase was also investigated by Blagojević et al. [73], revealing IC_50_ values ranging from 190 to 370 μg/mL. Additionally, Tenuta et al. [16] reported significant activity, with an IC_50_ of 30.40 μg/mL for the ethanol Soxhlet extract of fresh *C. mas* fruits, while other extracts exhibited values within the range of 54.5–312.4 μg/mL. Dzydzan et al. [74] assessed the impact on *α*-glucosidase, resulting in IC_50_ values of 28.5 and 25.7 μg/mL for yellow and red cornelian cherry fruits, respectively. According to the computational analysis, anthocyanins and ellagic acid emerged as the most effective inhibitors of *α*-amylase and *α*-glucosidase [73]. Among them, pelargonidin 3-robinobioside stood out as the most potent inhibitor, particularly exhibiting strong inhibitory activity against *α*-glucosidase [73]. Regarding pancreatic lipase inhibition, Świerczewska et al. [72] highlighted the ethanolic extract’s potential with an IC_50_ of 15.2 ± 3.9 μg/mL, while the aqueous extract exhibited an IC_50_ of 34.2 ± 4.2 μg/mL. Tenuta et al. [16] found substantial inhibition in certain extracts, such as hydroalcoholic maceration, displaying an IC_50_ of 74.3 μg/mL, and fresh fruit extract through the same method exhibiting an IC_50_ of 86.6 μg/mL.

For anti-acetylcholinesterase activity, Dzydzan et al. [74] reported that red and yellow cornelian cherry fruit extracts inhibited AChE by 70.1% and 58.4%, respectively, at 1 mg/mL. However, similar to our study, the extracts were weaker against AChE compared to neostigmine. For anti-tyrosinase activity, Natić et al. [75] found that cornelian cherry extracts displayed inhibition ranging from 21.75% to 74.23%. Furthermore, Nizioł-Łukaszewska et al. [76] explored iridoids from *C. mas* fruits, reporting that the iridoid water extract and iridoid water/ethanol extract exhibited up to two-fold higher inhibition than hydroquinone, reaching an inhibition percentage of 83.4% and 88.9%, respectively. These findings suggest diverse potential applications for *C. mas* fruit extracts in the management of metabolic and neurodegenerative diseases, but also in the food, cosmetic, and insect control industries.

### 4.5. Correlation Between Phenolic Classes, Multifunctional Bioactivities, and Study Limitations

Looking at the significant correlations reported, tyrosinase inhibition, often associated with skin-lightening and anti-melanogenic applications, showed strong correlations with various phenolic classes in *C. mas*, especially flavonols, phenolic acids, flavanols, and anthocyanins. This correlation suggests that these phenolic compounds might effectively contribute to the inhibition of tyrosinase, likely through mechanisms such as competitive binding to the enzyme’s active site. The broad efficacy across these classes also implies potential synergistic effects in mixed-phenolic environments, making *C. mas* extracts a valuable candidate for skin-care products aimed at hyperpigmentation issues. Regarding the DPPH radical scavenging assay, it represents a standard measure of antioxidant activity, and it was most significantly explained by the presence of flavonoids (both flavanols and flavonols) and phenolic acids. This strong correlation aligns with established knowledge that flavonoids and phenolic acids are highly effective in neutralizing free radicals due to their hydrogen-donating abilities. These findings highlight the potent antioxidant capacity of *C. mas* and support its application in health supplements or functional foods aimed at counteracting oxidative stress. Finally, the inhibition of amylase and glucosidase, enzymes involved in carbohydrate digestion, was uniquely correlated with the “other phenolics” category, including several phenolics exclusively correlated with the utilization of the UAE system. The capacity of these phenolics to inhibit these enzymes suggests that *C. mas* could help modulate postprandial blood glucose levels, offering a natural approach to glycemic control. This relationship points toward the potential use of these extracts in managing or preventing conditions like type 2 diabetes. These insights indicate that *C. mas* fruits’ phenolic profile offers multifunctional bioactivities relevant to skin health, antioxidant support, and glucose regulation. Each phenolic class’s distinct bioactivity also supports targeted extraction or formulation approaches, enhancing specific benefits based on the desired therapeutic application.

In addition to these multifunctional bioactivities, several limitations of the present study should be acknowledged. First, metabolite identification relied on untargeted UHPLC-HRMS and thus remains putative (MSI level 2), without confirmation through targeted MS/MS fragmentation or authentic reference standards. Second, although extractions were performed using biological material from independent batches, the final analysis was carried out on pooled samples and assessed in technical triplicates, which limits the evaluation of biological variability. Finally, the functional relevance of the observed antioxidant and enzyme-inhibitory activities was not assessed in cellular or in vivo models, and future studies incorporating targeted metabolomics and biological validation are required to substantiate the biochemical effects reported here [77].

## 5. Conclusions

This study provides the most comprehensive metabolomic characterization of *C. mas* fruits to date, integrating four extraction techniques (maceration, decoction, UAE, and EAE) with untargeted UHPLC-HRMS profiling, semi-quantitative class-level analysis, and multifunctional bioactivity assays. The study included two major novelties: (i) the first application of EAE to cornelian cherry fruits for phenolic recovery, and (ii) the broadest UHPLC-HRMS annotation reported for this species, with 342 tentatively identified metabolites, thus substantially expanding the known phytochemical diversity of cornelian cherry fruits. Extraction conditions markedly shaped metabolite composition: UAE yielded the highest levels of total flavonoids (433.6 µg eq./g) and phenolic acids (420.2 µg eq./g), whereas EAE selectively enriched hydrolyzed phenolics, reaching 1975.4 µg oleuropein eq./g, which is the highest quantity among all extraction techniques.

Beyond profiling, this study shows that cornelian cherry fruit extracts possess different bioactivities. They moderately inhibited enzymes involved in carbohydrate metabolism (α-glucosidase, α-amylase), lipid digestion (lipase), neuroprotection (acetylcholinesterase), and melanin accumulation (tyrosinase). Although these activities were weaker than those of pharmaceutical standards, cornelian cherry fruit extracts have the potential for applications in managing type 2 diabetes, obesity, neurodegenerative, and dermatological conditions.

However, it must be acknowledged that identified metabolites remain tentative (MSI level 2) and require validation through targeted MS/MS with authentic standards. Nonetheless, this study demonstrates that cornelian cherry fruits are much more complex in terms of their phenolic matrix than previously recognized, and that the extraction technique is a decisive factor shaping metabolite recovery and functional potential.

## Figures and Tables

**Table 1:** Phenolic composition of the different *C. mas* extracts, considering the different compound classes: cyanidin eq. (anthocyanins), catechin eq. (flavan-3-ols), quercetin eq. (flavonols), luteolin eq. (flavones and other remaining flavonoids), oleuropein eq. (other remaining phenolics), ferulic acid eq. (phenolic acids), resveratrol eq. (stilbenes). The results are expressed as mean values ± standard deviation (µg/g dry mass, *n* = 3). Superscript letters (^a,b,c,d^) within each row indicate significant differences as resulting from ANOVA and Duncan post hoc test (*p* < 0.05). **CME**—enzyme-assisted extraction extract; **CMF**—decoction extract; **CMM**—maceration extract; **CMU**—ultrasound-assisted extraction extract.

Phenolic Equivalents (Eq.)	Tentatively Identified Compounds	CME (µg/g)	CMF (µg/g)	CMM (µg/g)	CMU (µg/g)
Cyanidin eq.	Cyanidin-3-glucoside; Pelargonidin 3-*O*-arabinoside; Delphinidin 3-*O*-galactoside; Malvidin 3-*O*-galactoside; Peonidin 3-*O*-diglucoside-5-*O*-glucoside	48.6 ± 3.5 ^a^	49.7 ± 2.1 ^a^	63.6 ± 0.4 ^b^	62.8 ± 4.2 ^b^
Catechin eq.	(+)-Catechin; (−)-Epigallocatechin 3-*O*-gallate; Epicatechin 3’-*O*-glucuronide; 3’-*O*-Methylepicatechin	11.8 ± 2.6 ^a^	11.9 ± 2.2 ^a^	28.6 ± 0.9 ^b^	90.2 ± 1.4 ^c^
Quercetin eq.	Quercetin; Kaempferol 3-*O*-rhamnoside; Myricetin 3-*O*-galactoside; Isorhamnetin 4’-*O*-glucuronide	10.7 ± 0.2 ^a^	7.0 ± 0.1 ^a^	25.6 ± 1.5 ^b^	47.0 ± 5.4 ^c^
Luteolin eq.	Luteolin 7-*O*-glucoside; Apigenin 7-*O*-glucuronide; Baicalein; Chrysoeriol 7-*O*-glucoside; Sinensetin	222.9 ± 47.4 ^b^	196.3 ± 3.7 ^ab^	150.8 ± 27.9 ^a^	233.6 ± 1.6 ^b^
Oleuropein eq.	Caftaric acid; Pyrocatechol; Hydroxytyrosol derivatives; Oleuropein-type phenolic fragments; Secoiridoid-linked phenolics	1975.4 ± 35.1 ^b^	1506.5 ± 40.6 ^a^	1701.3 ± 94.9 ^ab^	1350.7 ± 30.3 ^a^
Ferulic acid eq.	Ellagic acid; Syringic acid; Caffeic acid; *p*-Coumaric acid; Ferulic acid; 3-Caffeoylquinic acid	104.4 ± 2.2 ^b^	46.0 ± 0.2 ^a^	263.1 ± 5.0 ^c^	420.2 ± 17.2 ^d^
Resveratrol eq.	*trans*-Resveratrol; Piceatannol; Dihydroresveratrol	107.9 ± 33.5	88.9 ± 1.7	107.6 ± 11.0	94.5 ± 8.0

**Table 2:** The in vitro anti-enzymatic potential of cornelian cherry fruit extracts obtained using various extraction methods. Statistical variances were assessed through the utilization of one-way ANOVA, followed by Tukey’s HSD post hoc test (*p* < 0.05). Distinct lowercase letters signify notable distinctions among cornelian cherry fruit extracts, while the presence of an asterisk (*) denotes a significant difference between the reference inhibitor and the assessed extracts. The presence of percentage (%) represents the inhibition activity of extracts at 4000 μg/mL. **CME**—enzyme-assisted extraction extract; **CMF**—decoction extract; **CMM**—maceration extract; **CMU**—ultrasound-assisted extraction extract.

	IC_50_ Value (μg/mL)				
Bioassay	CME	CMF	CMM	CMU	Reference Inhibitor
*α*-glucosidase Inhibition	151.42 ± 4.36 ^c^	80.61 ± 4.61 ^ab^	92.38 ± 8.1 ^b^	77.17 ± 4.80 ^a^	Acarbose: 48.15 ± 2.83 *
*α*-amylase Inhibition	233.85 ± 14.29 ^c^	85.54 ± 5.46 ^b^	76.47 ± 4.54 ^b^	52.54 ± 1.58 ^a^	Acarbose: 19.37 ± 1.12 *
Lipase Inhibition	1434.68 ± 99.58	55.40 ± 0.86%	52.24 ± 0.55%	54.80 ± 0.32%	Orlistat: 68.47 ± 2.98 *
Acetylcholinesterase Inhibition	280.81 ± 86.17 ^b^	140.07 ± 24.78 ^a^	118.43 ± 24.80 ^a^	134.76 ± 26.40 ^a^	Galantamine: 0.12 ± 0.01 *
Tyrosinase Inhibition	406.27 ± 91.26 ^a^	512.89 ± 71.52 ^a^	891.31 ± 128.11 ^b^	1184.17 ± 224.72 ^b^	Kojic acid: 0.11 ± 0.01 *

**Table 3:** Pearson’s correlation coefficients between the different phenolic classes and the bioactivity values measured. ns = not significant; * = *p* < 0.05; ** = *p* < 0.01.

	Anthocyanins	Flavanols	Flavonols	Other Phenolics	Phenolic Acids
AChE	−0.610 *	ns	ns	ns	ns
Amylase	−0.675 *	ns	ns	0.695 *	ns
Glucosidase	ns	ns	ns	0.738 **	ns
Lipase	0.597 *	ns	ns	−0.598 *	ns
Tyrosinase	0.757 **	0.852 **	0.925 **	ns	0.890 **
DPPH	ns	0.880 **	0.743 **	ns	0.673 *
FRAP	ns	ns	ns	−0.771 **	ns

## Data Availability

The original contributions presented in this study are included in the article/Supplementary Material. Further inquiries can be directed to the corresponding author.

## Supplementary Materials

The following supporting information can be downloaded at https://www.mdpi.com/article/10.3390/antiox14121419/s1. File S1: Excel file containing (a) and (b) the UHPLC-HRMS dataset with the annotated compounds; (c) the VIP discriminant compounds as a function of the different extraction methods.
